# Oral health in children with physical (Cerebral Palsy) and intellectual (Down Syndrome) disabilities: Systematic review I

**DOI:** 10.4317/jced.52922

**Published:** 2016-07-01

**Authors:** Montserrat Diéguez-Pérez, Manuel-Joaquín de Nova-García, Mª Rosa Mourelle-Martínez, Begona Bartolomé-Villar

**Affiliations:** 1 Dentist. Associate Professor. Department of Stomatology IV. School of Dentistry. Universidad Complutense de Madrid. Assistant Professor of the Department of Dentistry. School of Biomedical Sciences. European University of Madrid; 2 Stomatologist. Tenured Professor of Paediatric Dentistry. Department of Stomatology IV. School of Dentistry. Universidad Complutense de Madrid; 3 Stomatologist. Contract Professor, PhD. Department of Stomatology IV. School of Dentistry. Universidad Complutense de Madrid; 4 Stomatologist. Associate Professor, Department of Dentistry. School of Biomedical Sciences. European University of Madrid

## Abstract

**Introduction:**

Traditionally, patients with physical and/or intellectual disabilities presented greater oral pathology, owing to their condition and to other external factors. Improved social and health conditions make it necessary to update knowledge on their oral and dental health.

**Material and Methods:**

For this purpose, a bibliographic review was done regarding the state of oral health of children with these two types of disability, in comparison with a control group. Some of the guidelines of the PRISMA statement were taken into account. The ranking of the articles found is based on the modified Newcastle-Ottawa Quality Assessment Scale. The final number of articles evaluated was 14. Parameters such as dental caries, oral hygiene, gingival health, dental traumas, malocclusion and habits were considered.

**Results:**

There is no consensus among authors regarding dental caries, oral hygiene and gingival health. The different results obtained are due in part to the fact that the methodologies used were not the same. However, it has been noted that, when studying other parameters and regardless of the methodology employed, the results obtained are similar.

**Conclusions:**

Children with physical and intellectual disabilities constitute a group that needs early and regular dental care in order to prevent and limit the severity of the pathologies observed.

** Key words:**Oral health, dental caries, malocclusion, oral habits, dental trauma, oral hygiene, disabled child, cerebral palsy and Down syndrome.

## Introduction

People with physical (cerebral palsy, CP) or intellectual (Down Syndrome, SD) disabilities, as a result of the physical and intellectual deficiency which interferes with their normal functions, require more care and supervision in all their activities in life, including those related to their oral health. Traditionally, this group has been regarded as having more extensive and intense oral and dental pathology. Some specific factors (the disability itself, the treatments, lack of cooperation, etc…) and interpretations (lack of specialised centres and experienced professionals, neglect, lack of planning, etc…) have been used over the years to explain this situation. Fortunately, improvements in social and health conditions, improved quality of life and life expectancy, and greater access to medical resources and treatment, have led to an overall increase in the demand for dental care for disabled people. Providing an efficient response to this demand requires suitable training for professionals in a clinical environment without physical barriers, allowing easy access to this population so that they can receive treatment in accordance with their needs. What is needed, then, is exact knowledge of the dental situation of the different disabled populations. To this end, some publications have studied the oral and dental pathologies that are most prevalent in this group. From these studies, we can obtain a very up-to-date vision of this reality, which is useful for the correct planning of treatment services (1,2).

From this point of view, we have established the following goal.

To examine the available bibliography on the oral health of children with CP and DS, in order to determine whether there are differences between them and the general population as regards the state of their oral health.

## Material and Methods

The question that arises from the review is: do children with physical disabilities (cerebral palsy) or intellectual disabilities (Down syndrome) present greater oral and dental pathology than healthy children?

For the systematic review, we took into account some of the guidelines of the PRISMA statement:

1. Search strategy:

A search was done for scientific articles published in journals listed in the following databases: PubMed/ Medline, Scopus and Cochrane Library.

The keywords used were: “oral health”, “dental caries”, “malocclusion, “oral habits,” “dental trauma”, “oral hygiene”, “disabled child*,” to which were added the specific terms for the two disabilities, “cerebral palsy” and “Down syndrome.”

2. Criteria for selecting articles.

The search included original studies published between 2000 and 2015 in the databases mentioned (PubMed, Scopus and Cochrane), without language restriction, and which met the following criteria:

1. Study sample: children with CP or DS, more than 10 individuals between 0 and 18 years of age.

2. Control sample; healthy children.

3. Study variables: estimation of oral health, including at least one of the following; dental caries, oral hygiene, traumas and malocclusion.

Exclusion criteria, particularly those related to.

1. Study sample: sample size of less than 10 patients, age range of over 18 years;

2. Control sample: unhealthy children 

3. Bibliographic reviews, presentations to congresses, publishing houses, opinion articles or reports of isolated cases, etc.

-Extraction of data, variables used and presentation of the results

Two independent reviewers, each one covering one type of disability, did the selection of articles. In the first selection, any articles appearing in all three databases were excluded. Subsequently, potentially relevant articles were screened, and those that did not meet the selection criteria were excluded. The results of the variables analysed were reported separately, using a data collection table designed for that purpose.

-Evaluation of selected articles

A ranking table was designed, based on the Newcastle-Ottawa Quality Assessment Scale, with modifications ( Table 1). The information in each article was analysed in three principal categories, with subcategories that add nuances to the evaluation:

**Table 1:**
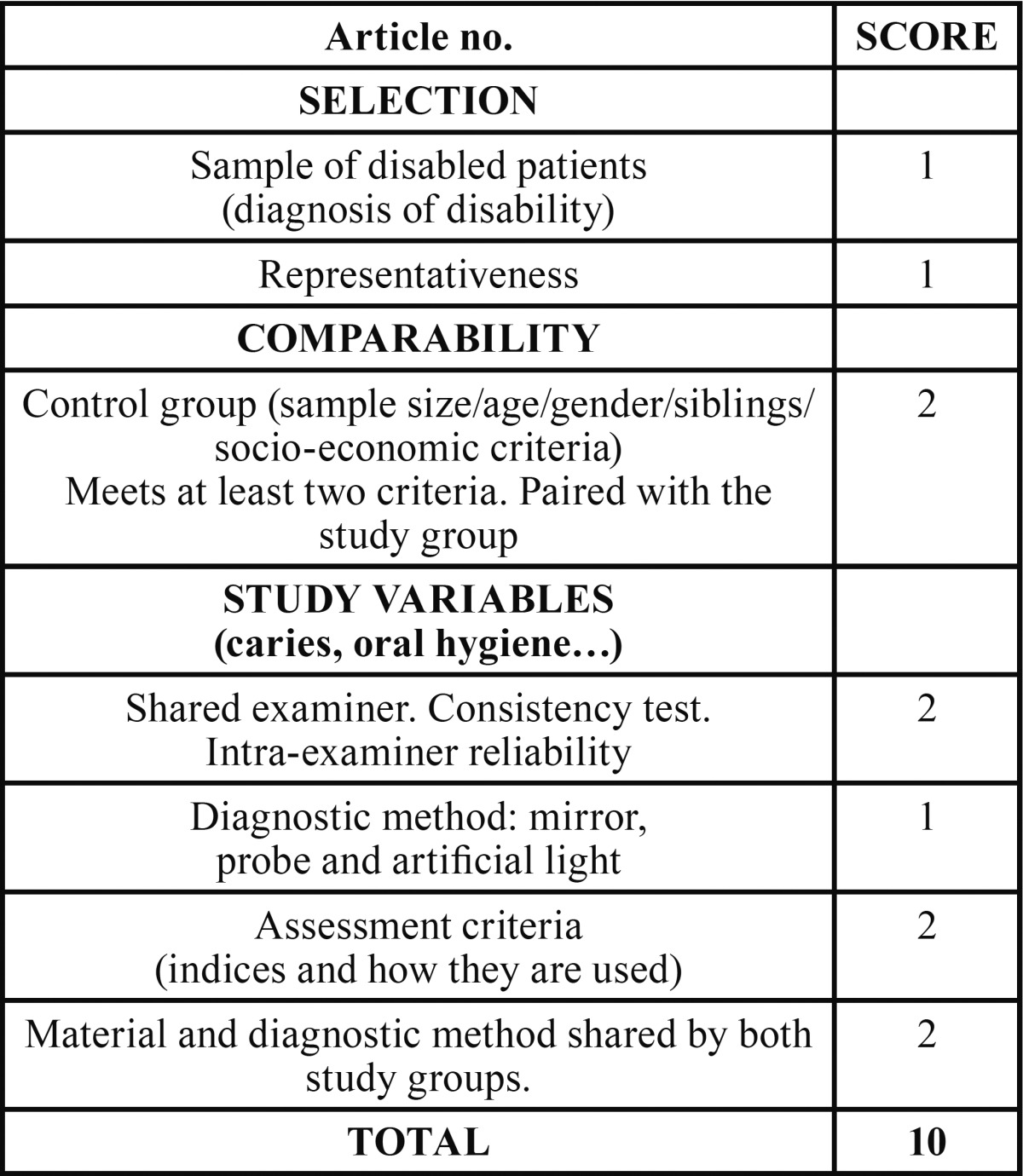
Table on assessment of selected articles.

1. Selection. Refers to the presence of the disabled group and the representativeness of the study sample.

2. Comparability. We considered whether the control group presented characteristics similar to those of the study group. A positive evaluation implies that at least two criteria have been met.

3. Variables studied. Regarding the oral health of the sample and the methodology of the study, information regarding the following was assessed.

- Characteristics of the examiner/s. Tests for reliability (intraexaminer) and consistency (inter-examiners).

- Methods for diagnosing the study variables. Common use in both samples (positive evaluation).

- Criteria for evaluating the variables used.

In order to be included, all articles had to have a minimum value of three points in the principal sections (1: CP or DS disability in a population of 0-18 years, 2: Comparison with a healthy control group, 3: Inclusion in the study of at least one of the variables selected). A score was assigned, which increased according to how the criteria included in each subsection were met.

## Results

**Figure 1:**
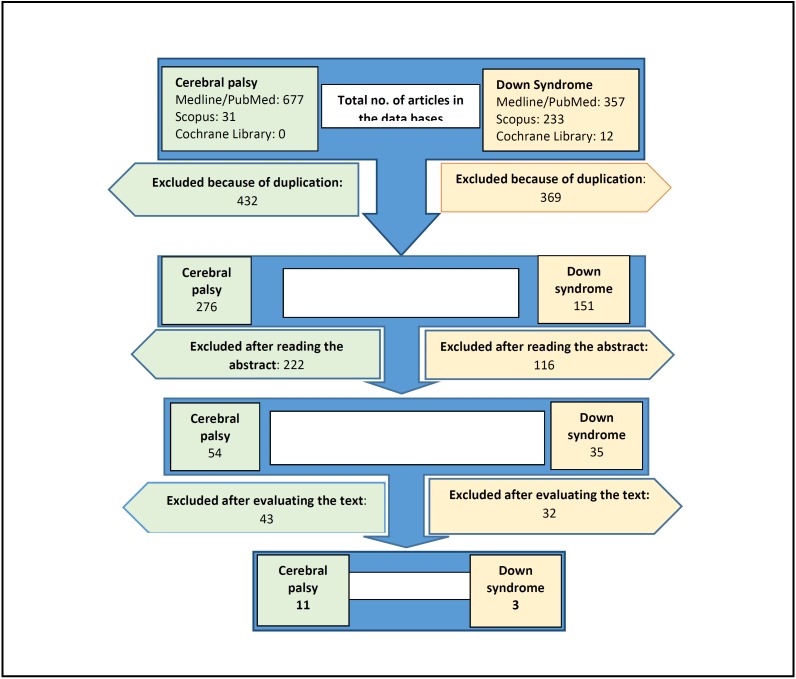
Flow diagram for the two reviews.

1.- Search results:

In the electronic search, a total of 1,310 articles were found. After successive exclusions reflected in the flow chart (Fig. 1) and after applying the selection criteria, the complete text of 54 articles on children with CP were read. Of these, 17 were excluded because they had a control group over 18 years of age; 22 because they had no control group; and four articles because they had no specific CP population (2) or an unhealthy control population (2). Regarding the studies of a population of children with DS, the complete text of 32 articles was read, of which 15 were excluded because the sample group was over 18 years of age; 12 because they did not have a control group and three because they included an unhealthy population in the control group. The final selection included 11 articles on patients with CP and three with DS.

2.- Study population:

A total of 1,796 patients were examined, of which 939 presented disability (CP or DS) and 857 were healthy (controls). The sample sizes varied between 18 children (3) and 103 children (4).

3. Intervention:

All the children received a dental examination, some of which were performed in dental clinics (4-9), or in the patient recruitment centre (10). The majority of the studies were done with the intervention of a single examiner (calibrated). The rest of the articles did not completely specify this information (3,11,12).

The majority of the clinical examinations were done with a tooth probe and mirror, although this is not always specified (6,9,10,13), and under conditions of artificial light in six of the articles. Only in the study by Batista were x-rays used to complement the clinical examination.

Regarding the methodology used to study the variables in our review:

-The incidence of dental caries tends to be studied according to WHO criteria, using the CAOD/CAO and caod/cao indices (4,5,8,10,12,14,15).

-The state of oral hygiene of these patients was analysed in 10 of the articles (3-5,7-10,13-15) using different plaque indices: the Green and Vermillion oral hygiene index (OHI) and the simplified (OHI-S) index, the Silness and Löe and the Podshadley and Haley plaque indices, the Silness and Löe gingival indices, the Massler (M-PMA) gingival inflammation index, the depth of probe (DP), calculus rate.

-The prevalence of dental trauma is evaluated in four articles, following the Andreasen criteria (11,13,15,16).

-Malocclusion is studied on the basis of different classifications, evaluating different parameters: the Angle classification (4,15), bite and overbite (13), crowding and anterior open bite (4).

-The presence of habits such as bruxism (8).

-Some articles include the study of variables not covered in our review: dental erosion or abrasion on the basis of O’Brien’s criteria (6,13,15), enamel defects or alterations (13,15) and other alterations in dental development (4); saliva parameters (15) and levels of *Streptococcus Mutans* and *Lactobacillus* (5); the incidence of dental-maxillary anomalies (10); delay in eruption (first permanent molar) (15); periodontopathogens (7), etc.

4. Results of intervention:

- Prevalence and experience of dental caries

Children with a physical disability (CP) seem to be more affected by dental caries; Botti *et al.*, 2010 find significant differences in mixed dentition (14); also, the frequency of extracted and filled teeth is significant in the study by Gržić *et al.* (12) and not significant for Alter *et al.* (10). Only Uraga-González *et al.* (15) observe a lower CAOD in children with CP (71% compared to 93%), with a similar cod index in both groups. In this case, the difference is statistically significant.

As for intellectual disability, the children with DS in the studies reviewed seem to be less affected by dental caries than the healthy population. Areias *et al.* (8) found a higher percentage of children without caries in the population with DS (78% compared to 58%). In the study by Al Habashneh *et al.* (4) they have a similar level of dental caries. There are no differences in the percentage of children without dental caries. Only male children with DS have significantly fewer caries than healthy children.

- Oral hygiene:

According to the studies by Ihtijarević-Trtak *et al.* (3), Botti *et al.* (5), Oliveira *et al.* (9), and Renan *et al.* (13), the group with a physical disability presents with worse oral hygiene. Statistically significant differences were found only in the articles by Oliveira *et al.* (9) and Renan *et al.* (13).

Similar oral hygiene habits (8) were found between DS and healthy groups (questionnaire). Clinical evaluation using the Green and Vermillion index (Oral Hygiene Index, OHI) shows, in the study by Morinushi *et al.* (7), a similar accumulation of plaque in the two groups. In the case of Al Habashneh *et al.* (4), they apply the simplified index (OHI-S), finding worse hygiene in children with DS (40% of the children had bad oral hygiene, compared to 23% in the control group).

- Gingival health

For analysis of the state of gingival health of the group with a physical disability (CP), the study by Alter *et al.* (10) shows better gingival health in the group with CP, as opposed to the study by Uraga-González *et al.*, which observed worse gingival health in the group with CP (57% compared to 43%) (15). Statistically significant differences were found only when the two groups were compared in the study by Alter *et al.* (10).

In children with an intellectual disability (DS), the studies showed worse gingival health than in the control group, with higher indices of gingival inflammation and also greater depth when probed (4),(7). Gingival health worsens with age (7).

- Dental trauma.

The group with a physical disability presented a greater frequency of dental traumas than the control group; all the authors agree on this point. According to the article by Batista *et al.* (11), the children with CP presented four times greater probability of dental trauma. This difference was statistically significant, as it was in the study by Uraga-González, *et al.* (15).

Other studies did not find significant differences (13,16). As for the type of trauma, the most frequent one was enamel fracture for the group with CP and crown fracture without pulp exposure for the control group. In both groups, the etiology of the traumas was falls (from the wheelchair, in the case of the group with disability). As for receiving treatment, more patients in the control group received treatment; in this case there were statistically significant differences between both groups (16).

In the children with DS, none of the articles selected referred to the prevalence of dental trauma.

- Malocclusion.

According to the study by Uraga-González *et al.*, the greatest number of type II and II malocclusions were found in the CP group (15) (33% compared to 15% for classes II, and 12% vs. 7% for classes III), this being a statistically significant difference. As for open bite and overbite, the children with physical disability presented a greater need for treatment of this malocclusion, this difference being statistically significant with respect to the control group (13).

Malocclusion is most frequent in the group of children with DS (69.9% compared to 40.8%), especially the presence of Class III (47.5% compared to 11.6%) (4). Open bite is also more prevalent in DS children (35.9% compared to 4.9%).

- Habits:

Children with physical and intellectual disabilities presented bruxism with significantly more frequency than healthy children: 22% compared to 0% for children with CP (15) and 23% versus 2% for children with DS (8).

Other variables studied in the articles reviewed revealed.

- Wear or abrasion, defects in enamel and other anomalies of dental development:

The first parameter (wear/abrasion) is significantly more frequent in the group with a physical disability (6,13). In children with DS, occlusal wear is also more frequent (36% compared to 11.7%).

As for defects in the enamel, they were more frequent in the group with CP (15) (23% compared to 13%), but the differences were not found to be significant.

The children with DS also presented a greater frequency of anomalies in dental development, such as hypodontia, with 51.5% compared to 4.9% (4).

- Lesions to the oral mucosa:

Children with CP show a greater frequency of lesions than healthy children, this being a statistically significant difference (13).

In the case of children with DS, a greater prevalence of fissured tongue was observed (56.3% compared to 2.9%) (4).

- Dental and maxillary anomalies (tooth and jaw anomalies)

The children with physical disabilities presented a greater number of anomalies than the control group, these being statistically significant differences (10).

- Others:

Botti *et al.*, 2002 (5) observed lower saliva flow rates in the CP group, in all the children with mixed or permanent dentition, except in girls with permanent teeth. This difference was statistically significant. The pH in the group with physical disability was lower than for the control group, this being a statistically significant difference. Girls with cerebral palsy and mixed dentition presented a greater number of *Streptococcus Mutans* colonies, this difference being statistically significant. Saliva incontinence was always greater in the group with CP and there were statistically significant differences between both groups in the study (15).

In the periodontopathogens test (BANA test) (7), although higher scores were obtained in the healthy control groups, in children with DS the scores were significantly greater among the older children.

Uraga-González *et al.* (15) found a greater delay in dental eruption in the group of children with CP, this being a statistically significant difference.

Other alterations of dental development (microdontia of maxillary lateral incisors) are more frequent in children with DS (4).

All the results obtained are shown schematically in  Table 2 and  Table 3.

**Table 2:**
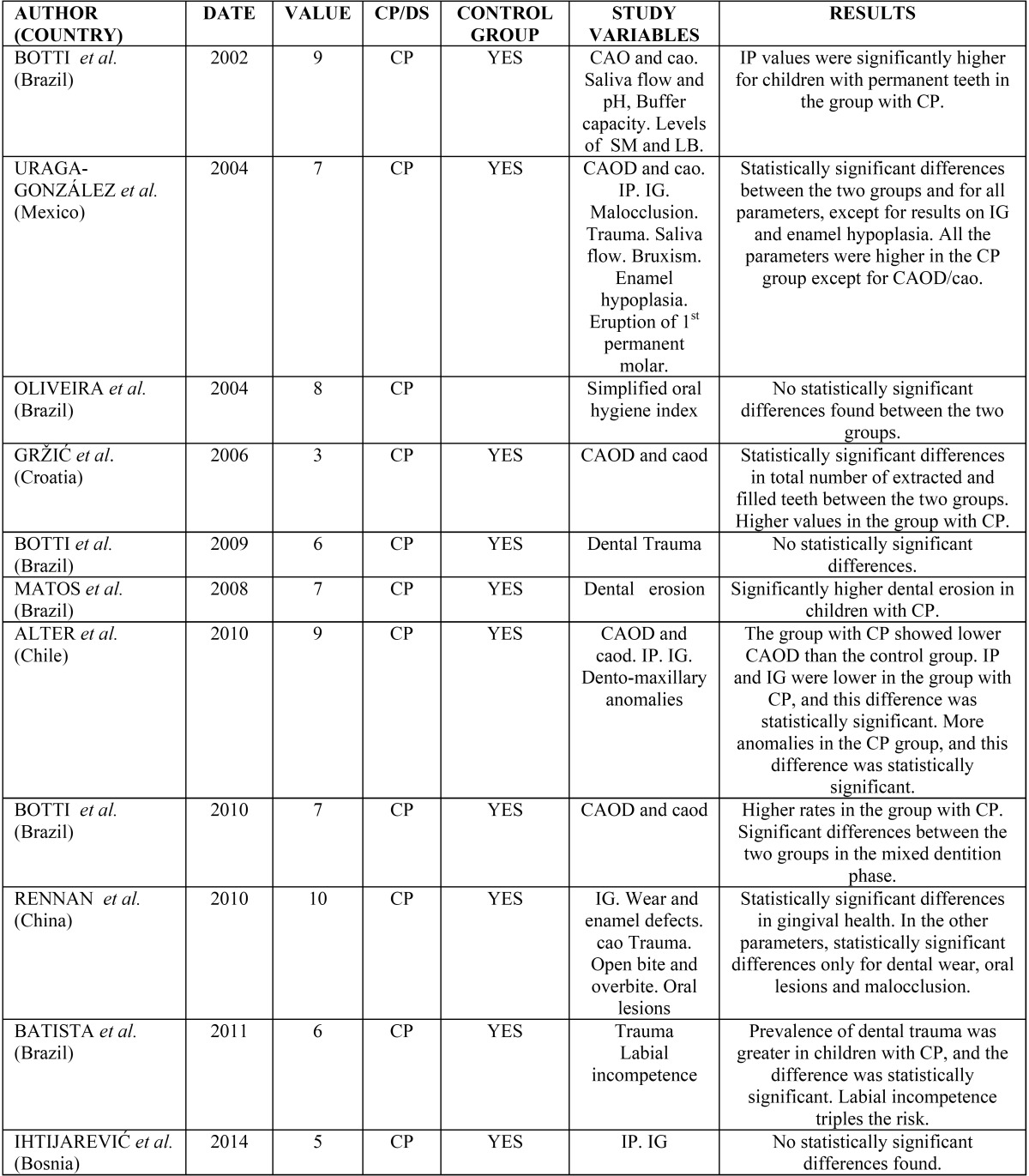
Results of the analysis for cerebral palsy.

**Table 3:**
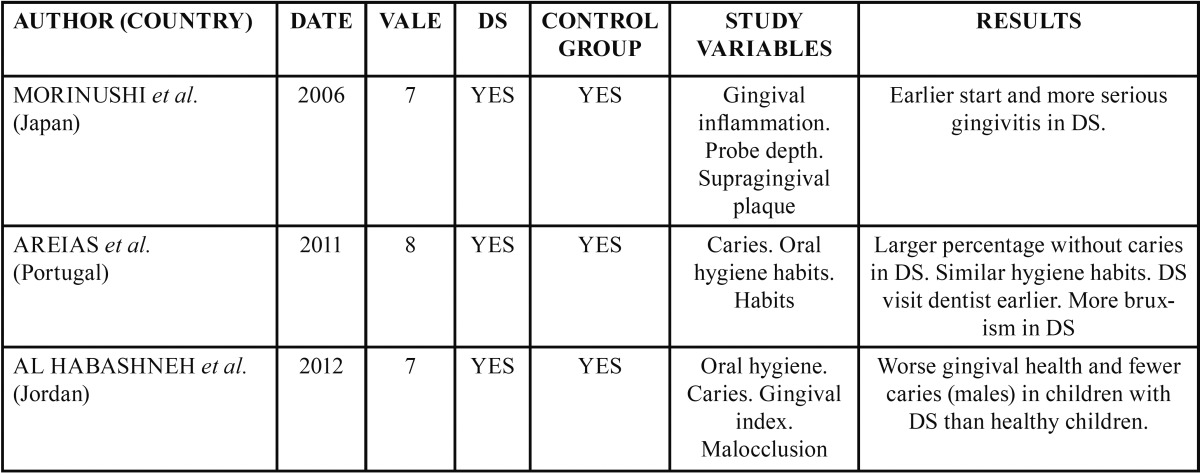
Results of analysis of articles for Down Syndrome.

## Discussion

There is a wide variety of research on the state of oral health of these disabled populations, but only a small percentage (1.55%) studied patients during childhood, with a control group and an appropriate sample size. On the basis of the scores obtained individually, the median value obtained was 7.0, which indicates high overall quality according to the ranking system utilized.

Regarding the total number of patients studied, there is no overall agreement regarding sample size, age range and gender between both populations. The largest age range studied was from 2-18 years (3) and the smallest, 30-73 months (9). The majority of the studies of physically disabled persons did not study the variables as a function of gender, only Botti *et al.* (5,14,16) and de Oliviera *et al.* (9). All the studies of intellectually disabled children included the study of gender (4,7,8).

The conditions for intraoral examination were not common to all the studies.

Regarding the incidence of dental caries, the children with intellectual disabilities (DS) were found to be freer from caries than their siblings (8) or with levels similar to those of the control groups (4). In children with physical disabilities (CP), lower values were also observed than in the CP group, with significant differences (15). In other studies, however, higher, statistically significant values were obtained in the group with CP (5,12,14).

When studying the level of oral hygiene, four studies found worse oral hygiene in the disabled population; three of them, in children with CP (3,5,9) and one in children with DS (4); in three of these, the differences were statistically significant (4,5,9). In the study by Areias *et al.* (8), the children with DS presented hygiene similar to that of their siblings. On the other hand, two studies of children with CP (10,15) found worse hygiene in the control group., with the difference being statistically significant only in the study by Alter *et al.* (10).

In general, children with disabilities presented higher gingival indices than the control groups, both those with physical disabilities (CP) (3,5,13,15) except in the study by Alter et al, although these authors believe this is due to continual supervision by carers (10), and those with intellectual disabilities (4,7). The differences reached significant levels in the studies by Alter *et al.* (10), and Renan *et al.* (13), and in some groups of children with DS (4,8).

Dental traumas were more frequent in the population with CP, with consensus among authors on this point (11,13,15,16). There were significant differences for Batista *et al.* and Uraga-González *et al.* (11,15).

Children with disabilities, both physical (CP) (15,13) and intellectual (4), were more affected by malocclusion. In both groups, the differences tended to be significant and malocclusion more prevalent, with classes II and open bite in children with CP and classes III in children with DS (4).

Children with disabilities presented greater wear or dental abrasion. In the case of children with CP (6,13,15) there were significant differences in the studies by Matos *et al.* (6) and Renan *et al.* (13).

Also, among children with DS (4), habits that give rise to these dental alterations (bruxism) are more prevalent than in their siblings (8).

With respect to the other variables studied, both the children with CP and those with DS presented a larger number of anomalies in dental development and delayed eruption (4,8,10,15), with the differences being, in general, statistically significant.

## Conclusions

With regard to the variables studied (dental caries, oral hygiene, gingival health, trauma and malocclusion) and the review criteria established, we consider that, in children with physical (CP) and intellectual disability (DS):

-The incidence of dental caries in children with CP is somewhat greater than in the control groups and similar, or lower, in children with DS.

-Oral hygiene is in general poorer than in the control groups;

-Gingival health is notably worse than in the control groups, above all in children with DS.

-Children with CP have a higher prevalence of dental traumas and

-Children with DS a greater frequency of habits (bruxism).

Additionally, other anomalies in dental development are more common in them, such as delayed eruption and the presence of wear and abrasions.

For all these reasons, they constitute a group that should receive early and regular dental care, in order to prevent and limit the severity of the pathologies observed.
